# Experimental, Numerical, and Analytical Study on The Effect of Graphene Oxide in The Mechanical Properties of a Solvent-Free Reinforced Epoxy Resin

**DOI:** 10.3390/polym11122115

**Published:** 2019-12-16

**Authors:** Sergio Horta Muñoz, María del Carmen Serna Moreno, José Miguel González-Domínguez, Pablo Antonio Morales-Rodríguez, Ester Vázquez

**Affiliations:** 1 Escuela de Ingeniería Industrial y Aeroespacial de Toledo, Instituto de Investigación Aplicada a la Industria Aeronáutica (INAIA), Departamento de Mecánica Aplicada e Ingeniería de Proyectos, Universidad de Castilla-La Mancha, Av. Carlos III, Real Fábrica de Armas, 45004 Toledo, Spain; mariacarmen.serna@uclm.es; 2 Group of Carbon Nanostructures and Nanotechnology, Instituto de Carboquímica (ICB-CSIC), C/ Miguel Luesma Castán 4, 50018 Zaragoza, Spain; 3 Instituto Regional de Investigación Científica Aplicada (IRICA), Av. Camilo José Cela s/n, 13071 Ciudad Real, Spain; 4 Escuela Técnica Superior de Ingenieros Agrónomos de Ciudad Real (ETSIA), Departamento de Producción Vegetal y Tecnología Agraria, Universidad de Castilla-La Mancha, Ronda de Calatrava 7, 13071 Ciudad Real, Spain; pablo.morales@uclm.es; 5 Department of Inorganic, Organic Chemistry and Biochemistry, Faculty of Science and Chemical Technologies, Universidad de Castilla-La Mancha, 13071 Ciudad Real, Spain; ester.vazquez@uclm.es

**Keywords:** polymer-matrix composites (PMCs), nano composites, mechanical properties, homogenized section technique, graphene oxide (GO), finite element method (FEM), scanning electron microscopy (SEM), digital image correlation (DIC)

## Abstract

This paper presents a methodology for manufacturing nanocomposites from an epoxy resin reinforced with graphene oxide (GO) nanoparticles. A scalable and sustainable fabrication process, based on a solvent-free method, is proposed with the objective of achieving a high level of GO dispersion, while maintaining matrix performance. The results of three-point bending tests are examined by means of an analytical technique which allows determining the mechanical response of the material under tension and compression from flexural data. As result, an increase of 39% in the compressive elastic modulus of the nanocomposite is found with the addition of 0.3 wt % GO. In parallel, we described how the strain distribution and the failure modes vary with the amount of reinforcement based on digital image correlation (DIC) techniques and scanning electron microscopy (SEM). A novel analytical model, capable of predicting the influence of GO content on the elastic properties of the material, is obtained. Numerical simulations considering the experimental conditions are carried out. the full strain field given by the DIC system is successfully reproduced by means of the finite element method (FEM). While, the experimental failure is explained by the crack growth simulations using the eXtended finite element method (XFEM).

## 1. Introduction

The discovery of different carbon nanoforms, such as fullerenes and carbon nanotubes (CNT), has opened the door for the development of new composite materials with improved mechanical properties [1,2,3]. Above all these carbon derivatives, graphene holds the highest expectations, not only due to its outstanding mechanical properties, but also based on its applications in fields, such as medicine, electronics, or the transport industry [4]. Although, these materials possess the required properties, they may not find their implementations in many of these areas, due to the existence of optimized technological alternatives at a lower cost [1,4]. For this reason, it is necessary to deepen our scientific understand of these materials, in order to optimize their use for the desired applications and establish a quality, economica, and scalable manufacturing process.

Regarding the mechanical properties of graphene, it is known as the one of the most resistant and stiffest material, with values of tensile strength ~100 GPa and Young’s modulus of ~1 TPa, surpassed only by both diamond and carbine. In spite of these high values, the real performance of graphene-based composites is still orders of magnitude below those values [5,6]. Additionally, it is considered the thinnest two-dimensional crystal in nature, with a thickness equivalent to a carbon atom and formed exclusively by carbon hexagonal arrangements. This structure is very difficult to achieve because, during the production process, defects and imperfections could appear. Nevertheless, these imperfections can give rise to graphene derivatives with lower production costs that present advanced applications [6,7,8].

Specifically, graphene oxide (GO) has raised great interest due to the presence of functional groups, which allow an easier integration with different compounds [7,8,9,10,11,12,13,14,15]. GO, unlike graphene, is an insulating, hygroscopic, and hydrophilic material with a Young’s modulus of ~200–300 GPa [16,17,18]. Although, the elastic modulus of GO is one order of magnitude lower than the pristine graphene, GO interacts better with other materials (for example, with the matrix of polymer-based composites), due to its rich diversity of native oxygen functional groups, and also because of its chemical versatility, which allows its tailored functionalization. This implies an improvement of effective properties of the resultant composite due to the enhancement of the stress transfer [7,19,20,21,22,23,24,25].

In the specialized bibliography there is a widespread agreement that dispersion and interface interaction are key factors for property enhancement in nanocomposites [8,21,22,23,24]. Tang et al. [24] conducted an interesting study about the effect of dispersion, where the addition of reduced GO (rGO) was compared with, and without, ball mill mixing. The rGO reinforcement leads to better mechanical properties when it is highly dispersed, thereby enhancing the fracture toughness.

Many advances have been made recently in the field of Polymer Matrix Composites (PMCs) reinforced with graphenic materials for structural applications [8,26,27,28,29,30]. Bortz et al. [26,27,28] demonstrated a significant fracture toughness and fatigue life improvement of an epoxy system, i.e., a Bisphenol A/F diglycidil ether (DGEBA/F) blend, through the addition of GO sheets previously suspended in acetone. The Young’s Modulus of the neat epoxy was enhanced by 6% and the tensile strength was increased by 13% when 0.5 wt % GO was added.

Another method for improving the interaction of the GO with epoxy matrices was developed by Li et al. [22], which consisted in both reducing and functionalizing with dopamine, followed by sonication in the epoxy monomer, Bisphenol A diglycidil ether (DGEBA). The results obtained in this study proved that functionalized GO performs mechanically better than untreated GO, improving the tensile and flexural moduli and strengths at 0.2 wt % GO loading.

The aforementioned methods combine the use of surfactants, solvents, reduction processes or chemical functionalization. For instance, Bao et al. [31] developed a Functionalized Graphene Oxide (FGO) by surface modification with hexachlorocyclotriphosphazene (HCTP) and glycidol, via in situ thermal polymerization. In the epoxy preparation, the authors added a solvent, namely tetrahydrofuran (THF). The use of solvent improves the dispersion of the reinforcement in the matrix, but its presence in the resultant material could mean a loss in some properties. To avoid this, the mixtures are usually subjected to long cycles in vacuum oven, which can be also detrimental to mechanical properties [23,32].

In summary, these studies provided an approach for the chemical interaction between reinforcement and matrix, without sufficient deepening into the mechanical behavior of these nanocomposites. Furthermore, few observations about compressive properties of PMCs have been performed. In order to take advantage of this nanostructure in real world applications, it is necessary to develop models which allow the prediction of the effective mechanical response of the resulting material and the final effect of GO on the set of mechanical properties. In this work, analytical equations, based on Continuum Mechanics, are proposed in order to analyze in detail the tensile and compressive mechanical response under flexural testing. This analysis is corroborated by means of numerical simulations based on the finite element method (FEM) and eXtended finite element method (XFEM). The importance of the process of obtaining the composite must be emphasized, since there is a generalized use of solvents and aggressive dispersion methods, i.e., sonication. The manufacturing steps proposed in this work avoid the use of solvents and aggressive mixing methods, leading to a scalable and environmentally friendly process.

## 2. Materials and Methods

### 2.1. Materials and Specimen Preparation

The specimens were fabricated by reinforcing an epoxy commercial system with GO and using graphite for comparison. In particular, the work includes the production of neat resin, resin reinforced with GO in four different percentages and resin reinforced with 0.3 wt % graphite, as listed in Table 1.

The epoxy matrix, a low temperature curing system with the commercial name of Gurit Ampreg 21, was kindly provided by Vestas Blades (Daimiel, Spain). This system is based on a blend of monomers DGEBA/F, and a highly reactive hardener, which according to the manufacturer [33], consists mainly of triethylenetetramine (TETA) and minor amounts of other organic compounds (namely 2-Piperazinoethylamine and 4-tert-Butylphenol). This resin, which is of low viscosity, provides ease of manufacture, since the curing is performed at room temperature. Although, a subsequent post-cure is recommendable to improve the mechanical properties.

The GO was supplied in the form of nanoplatelets by Grupo Antolin (Burgos, Spain), with the trademark GRAnPH [34]. This reinforcement is obtained by the application of a modified Hummers method to carbon nano-fibres, following the procedure detailed in [35]. The physicochemical characterization of GO is detailed in [36]. Some preliminary tests of nanocomposite preparation with the GO, as supplied, revealed that achieving even a minimal level of dispersion in the absence of solvents is impossible, despite the application of harsh ultrasounds. Therefore, an alternative procedure for improving the affinity of GO was developed. This process consists of the GO dispersion in water, various cycles of centrifugation and redispersion and, finally, freeze-drying, which manage to remove acid traces of the material. In previous works, it has been stated that freeze-drying of graphene derivatives is a powerful way to efficiently disperse them in many media [37].

Graphite powder was integrated in the epoxy system as supplied by Bay Carbon Inc. (SP-1 grade). Graphite is used in this study as a control experiment in order to compare the mechanical response of the nano-reinforced epoxy with a non-dispersed micro-reinforcement of analogous morphology.

**Figure 1:**
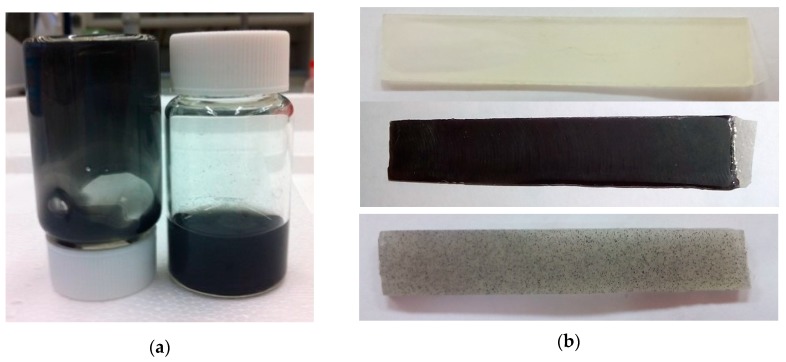
Specimen manufacturing: (**a**) Graphene-oxide (GO)-epoxy dispersion; (**b**) Neat resin, 0.3wt % GO- and graphite-reinforced specimens for bending testing.

For the preparation of the composite, an in-situ polymerization process was followed. The GO or graphite powder is added to the liquid monomer (in the convenient amount to reach the desired final wt %) and is dispersed by stirring at 60°C for 30 min. For that, the blend was placed in a glass vial (Figure 1a) and the stirring process is performed using a hot plate magnetic stirrer, which was set at about 120 rpm. Afterwards, low vacuum is applied for 5 min in order to degas the air retained in the stirring process, obtaining a liquid blend (Figure 1a). Subsequently, the blend is left to cool down to room temperature and the curing agent is incorporated in the proportions stipulated by the manufacturer, i.e., a monomer/hardener weight ratio equal to 100/33, producing small batches around 12 g. Further stirring at ambient temperature for 5 min is necessary in order to disperse the hardener. The final mixture is poured into PTFE molds (being the choice of this material because of its nonadherence properties) with the shape of the sample specimens, such as those shown in Figure 1b. Afterwards, they are allowed to cure at ambient temperature (at 18 °C) for at least 16 h, and then, transferred to an oven in which a post-curing (at 50 °C for 8 h) was applied.

### 2.2. Three-Point Bending Test

In order to study the influence of the nanoreinforcement on the mechanical properties, three-point bending tests are applied to the developed materials. This experimental technique involves the application of a load over the midsection of a pinned-pinned beam.

The results of three-point bending testing can be used in determining the tensile and compressive response of the material that develops the data analysis with the homogenized section technique [38,39,40]. This methodology has been chosen, instead of the standard uni-axial testing, because of the reduced dimensions of the specimens. In other words, it leads to a more economical and sustainable determination of mechanical properties.

**Figure 2:**
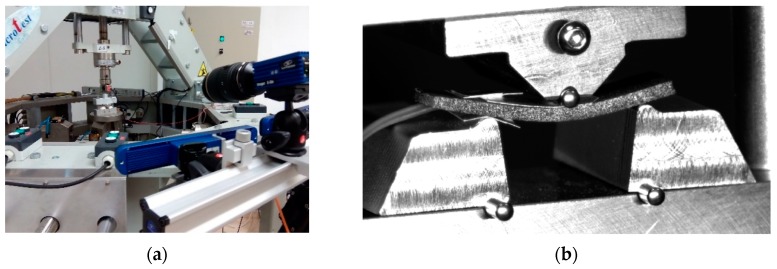
Experimental facility: (**a**) Electromechanical testing machine and digital image correlation (DIC) system; (**b**) Specimen image obtained by DIC system during testing.

**Figure 3:**
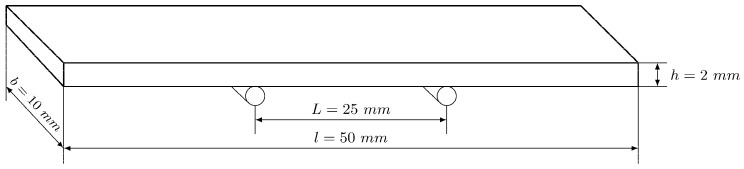
Bending specimen shape and dimensions.

The three-point quasi-static bending tests have been performed in a MICROTEST MAEFH electromechanical testing machine (Figure 2a) with a 5 kN load cell, setting a constant displacement rate of 0.3 mm/min. Prismatic specimens are used with dimensions of 10 mm width, 2 mm thick and 50 mm length, supported in 25 mm span, as schemed in Figure 3. At least five repetitive tests for each proposed material are obtained. A three-dimensional (3D) Digital Image Correlation (DIC) system, LaVision StrainMaster, and two cameras, LED illumination and controller, are used to obtain the full strain field at the lateral side of the specimen (Figure 2a). A commercial software called Davis is applied for the acquisition and post-processing of data. The procedure involves the acquisition of images at constant frequency, having the specimen a speckle pattern obtained by means of black and white spray painting (Figure 2b). The images are discretized into small subsets of pixels, and the cross-correlation algorithm tracks the pattern of gray level within each subset. The local displacement is recorded where the pattern matching is maximized. Repeating this across the entire image for all subsets yields a full field map of displacements, deriving from them the strains.

#### Homogenized Section Technique

**Figure 4:**
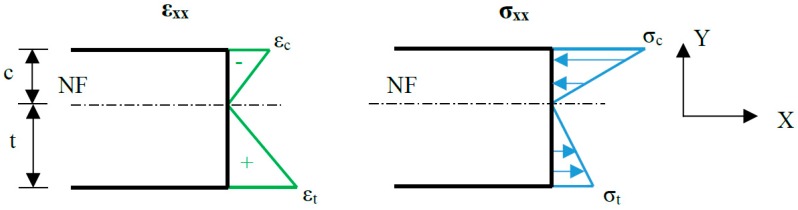
Normal strain and stress distributions on the cross-section under bending moment with *E_t_* ≠ *E_c_*.

This analytical methodology, based on previous works with Carbon Fiber Reinforced Polymer (CFRP) laminates [38,39,40], considers the different elastic response of the material under tensile and compressive loads. Polymers and CFRP composites exhibit usually higher values of strength and stiffness under compression. Therefore, the stress and strain distributions caused by bending moment are asymmetrical, as the neutral fibre (NF) deviates at a certain distance *d* regarding the mid-height plane of the specimen, as schematized in Figure 4. By means of the horizontal force equilibrium, it is possible to obtain the Equation (1) that relates the tensile and compressive elastic moduli, *E_t_* and *E_c_*, with the thicknesses of the regions submitted to tension and compression, *t* and *c*, respectively.

(1) $$n=\frac{E_{c}}{E_{t}}={\left(\frac{t}{c}\right)}^{2}$$

Based on this methodology developed in [38], expressions for the maxima stresses either under tension or compression can be written as function of the elastic moduli ratio *n* (Equations (2) and (3)):

(2) $$\sigma_{t}=\frac{3PL}{4bh^{2}}\frac{1+\sqrt{n}}{\sqrt{n}}$$

(3) $$\sigma_{c}=-\frac{3PL}{4bh^{2}}\left(1+\sqrt{n}\right)$$

In these equations, *P* represents the applied load in the center of the specimen, *L* is the support span, *b*, and *h*, are the width and thickness of the specimen, respectively.

Due to full field strain acquisition by means of the DIC equipment, the values of *t* and *c* can be determined experimentally and, therefore, the previous equations can be applied to the analysis of the three-point bending tests in order to determine the different elastic moduli.

### 2.3. Compressive Testing

In order to corroborate the properties measured by means of the three-point bending tests, compressive testing is performed by applying the recommendations of the standard ASTM D695 [41]. Cylindrical specimens with 20 mm height and 10 mm diameter are placed between compression plates and pressed under a rate of 0.5 mm/min to ensure a quasi-static application of load. As in bending testing, a minimum of five tests are performed, depicting stress-strain plot and obtaining mean and standard deviations for the elastic modulus and compressive yielding strength.

### 2.4. Numerical Simulations

Numerical models are created in order to support and validate the experimental results obtained with the flexural tests for the neat epoxy. Two different procedures are applied: Static linear elastic analysis by means of the FEM, and a crack-growth non-linear simulation using the XFEM. Both analyses are performed using the commercial software Abaqus 2018.

A two-dimensional (2D) plane stress model has been developed using the lateral dimensions of the prismatic specimen previously described. The element chosen to simulate the bending problem is the CPS8R, an eight-node biquadratic plane stress quadrilateral with reduced integration. The geometry is split in two areas according to the experimental position of the NF. This geometrical division allows assigning different tensile and compressive isotropic material properties, using the moduli measured experimentally. The boundary conditions are set according to the three-point bending test procedure, constraining the vertical displacement in both ends of the specimen and applying a vertical displacement on the top center of the coupon.

The initial static analysis allows to verify the validity of the analytical model to reproduce the deviation of the NF and, therefore, obtain asymmetric stress/strain distributions. Furthermore, the linear simulation provides displacement contour plots that are directly comparable with those obtained experimentally by the DIC system. In order to verify the validity of the numerical model, a sensitivity analysis is also carried out on the mesh of the elastic linear simulation. The procedure consisted of verifying the convergence of the results according to the element size. This analysis results involved the use of an adaptive mesh, with a bias from the NF to the exterior fibres, varying the size from 0.02, to 0.2 mm, respectively.

Extending the scope of the numerical model through FEM, the implementation of a material failure model is pursued, emphasizing the prediction of the initiation and evolution of the cracks. However, traditional implementation of FEM requires the creation of an initial crack and adaptive meshing. A recent alternative is the XFEM formulation [42,43]. The interpolation functions of displacements, on which the standard FEM are based, are enriched with discontinuous functions, which allow the collection of singularities close to the crack edge [44]. In order to define the crack initiation and propagation criteria, a traction-separation criterion is defined. Amongst the different parameters of the numerical model, the strength of the material, given in Table 2, comes from the maximum tensile stress measured in the three-point bending testing. This is based in the hypothesis of a tensile initiated failure, as it is expected to happen in most polymeric materials. Experimental results shown in next chapter sustain this hypothesis.

Regarding the crack evolution modelling, the experimentally observed failure is mainly brittle, with a rapid propagation of the crack, generating a single fracture plane with a crack initiated by tension and instantaneously propagated in mode I. This is corroborated by experimental evidences via a completely sudden drop in applied force and the microscopic examination of the fracture surfaces. The expected effect on the simulation in relation to the progressive release of energy during crack propagation is negligible and it is therefore estimated that the ultimate strain will be of the order of 1% more than the failure initiation strain. This approximation is also found in previous works [43]. Under this approach, the energy dissipated per unit volume in mode I can be calculated as stated in Equation (4). Finally, the mechanical properties of the epoxy resin used in the simulations are shown in Table 2.

(4) $$G_{I}^{c}=\frac{0.01\sigma_{f}\varepsilon_{f}}{2}$$

## 3. Results and Discussion

### 3.1. Three-Point Bending Testing

The 3-point flexural tests allow the characterization of tensile strength and both tensile and compressive stiffnesses of the material. The application of the DIC technique allows to obtain the distribution of normal strains through-thickness, so that it is straightforward to identify the difference in tensile and compressive behaviour. The methodology used to achieve this consists in obtaining the strains in the subsets arranged along the thickness, obtaining the strain value in various precise positions. Assuming linear response of the material and small strains and displacements, it is direct to perform a linear regression of the data, and thus to obtain the position of the NF. Consequently, the size of the areas under tension and compression, *t* and *c*, can be computed at any instant of the test. Additionally, the value of *n* is derived from Equation (1). It should be noted that DIC processing software considers the deformation of the specimen, and therefore the rotation of the section due to the rotational bending effects.

**Figure 5:**
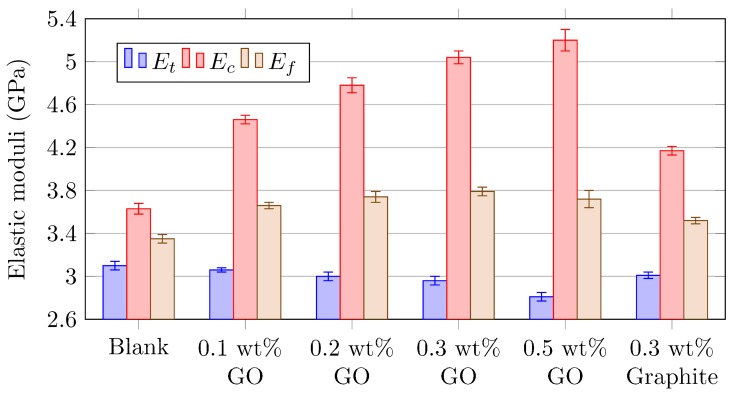
Bar chart showing evolution of tensile, compressive and flexural elastic moduli measured via bending testing.

In this way it is possible to obtain the maximum stresses corrected with the deviation of the NF, applying Equations (2) and (3), collecting the maximum values in Table 3. Besides, the elastic moduli calculated as the slope of the stress-strain evolution are listed in Table 4 and charted in Figure 5.

In view of previous results, some remarks should be made. Clear trends due to GO reinforcement are observed. On an overall basis, tensile properties tend to a slight decrease with the increment of reinforcement, while compressive properties show a notorious increase. As a matter of example, the 0.3% GO-reinforced epoxy results in an average increment of 39% of compressive elastic modulus, while tensile modulus is decreasing a 5% and ultimate tensile strength is reduced by 14% with regard to blank epoxy. This inference results promising for fibre-reinforced composite material, since the matrix has a greater relevance supporting compressive loads, while the fibres provide the tensile strength. Therefore, it is expected that reinforcing the thermoset matrix with GO could lead to a better global behaviour of the resulting composite.

Moreover, results for tensile strength and elastic moduli in Table 3 and Table 4 for the higher reinforcement-loaded case (0.5 wt % GO) show an important change in trend with regard to lower fractions of reinforcement. Therefore, extrapolation of results for reinforcement contents over 0.5% should be experimentally studied, expecting a degradation of properties caused by a saturation of the epoxy system, which is usually reflected in form of aggregates. This hypothesis agrees with many other experimental evidences found in literature [19,22,23,29,30,31,45], where other authors point out the inefficiency of high amount of nano-reinforcements.

In parallel, following the same manufacturing process, the inclusion in the blank resin of 0.3% graphite has been studied for comparison purposes. The results for this scenario lead to some interesting conclusions. On one hand, tensile modulus exhibits a lower detriment in relation to same amount of GO reinforcement. On the other hand, tensile strength shows a significant reduction as graphite flakes are bigger in size and generate stress concentrations, promoting a premature failure of the material. In addition, graphite absence of functional groups reduces the chemical interaction with the polymer, obtaining a physical reinforcement with a lower load transference. This idea is corroborated through visual inspection of the resultant material, as graphite aggregates can be observed under the naked eye (Figure 1b).

**Figure 6:**
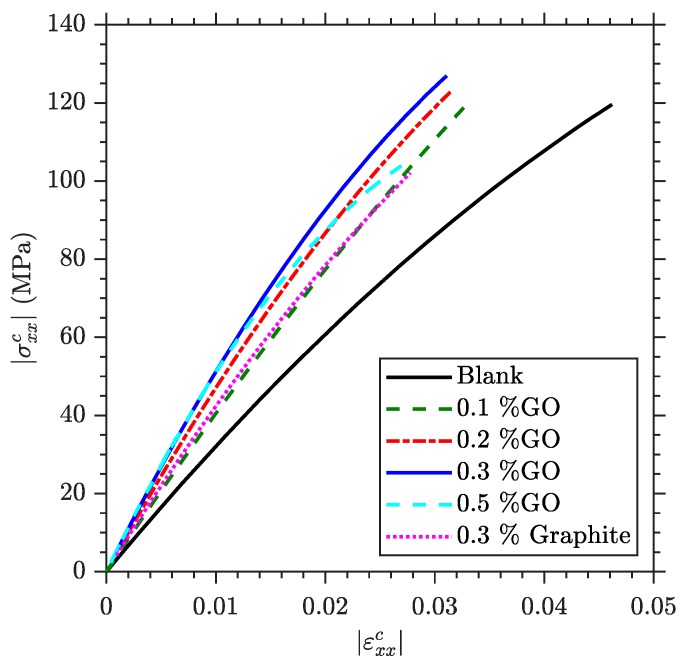
Stress-strain plots obtained from three-point bending tests at the midspan upper face.

For a better comprehension of the effect of reinforcements on the calculated mechanical properties, Figure 6 shows the stress-strain curves obtained at the upper fibre of the mid-section of the specimen, in other words, in the point of maximum compressive stress and strain. For ease of comparison, the absolute value of the compressive stress and strain has been plotted. Figure 6 highlights the increase in the maximum compressive stress and stiffness when increasing the percentage of GO. In addition, the presence of GO reduces the maximum strain and produces a more linear behaviour. It should be noted that [45] shows a very similar trend for the tensile stress-strain curves of a material reinforced with graphene nanoribbons, presenting a stiffening effect of the reinforcement, until reaching a saturation threshold at which there is a decrease in both modulus and strength.

Nevertheless, the reinforcement with GO should not be understood as what happens in a traditional composite, in which the resultant combination of components produces mechanical properties depending on volume fraction and reinforcement shape, as reflected by Rule of Mixtures and Halpin-Tsai model. These models predict a linear relation between elastic modulus and volume fraction of reinforcement, and some authors have tried to correlate experimental results with these factors by means of some arbitrary parameters [45,46]. What it is actually observed on this kind of nano-reinforced material, in which there is an expectedly strong filler-matrix interaction due to the affinity of GO functional groups and the epoxy, could not be related to physical-based equations for composites. In other words, the solvent-free combination of polymer and our processed GO is leading to a different composition of material with new behaviour.

**Figure 7:**
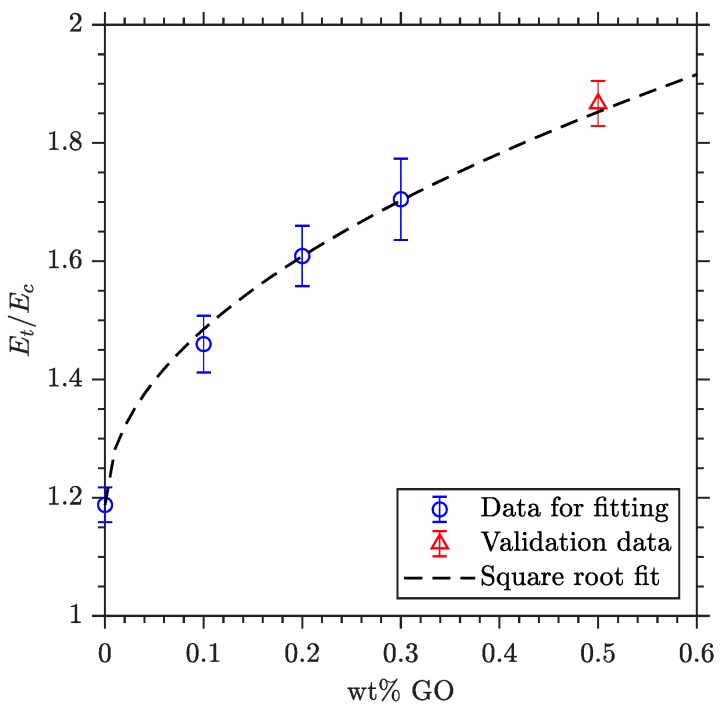
Fitting of evolution of moduli ratio with reinforcement load.

The results obtained for the value of *n* as a function of the studied GO percentages shown a predictable trend. As the analytical model applied relates the square of the thicknesses of the tensile and compressive loaded regions (*t* and *c*) with the corresponding elastic moduli ratio (Equation (1)), a similar relation is proposed to study the dependence of these parameters regarding the reinforcement fraction. A parallel application of this approach is found in related literature [47]. Figure 7 illustrates the result of the proposed analytical model, in which the experimental results obtained for the 0.1%, 0.2% and 0.3% GO reinforced specimens were used as input data for the least squares regression. Then, the analytical expression given by Equation (5) is obtained, which is applicable to predict the tensile-compressive ratio dependence on the amount of reinforcement in the polymer of study. Experimental result for 0.5% GO reinforcement is also plotted in Figure 7 as a matter of validation of the good agreement of the model up to this percentage.

(5) $$n=n_{0}+a\cdot \sqrt{wt\%GO}=1.1878+0.94\cdot \sqrt{wt\%GO}$$

### 3.2. Compressive Testing

**Figure 8:**
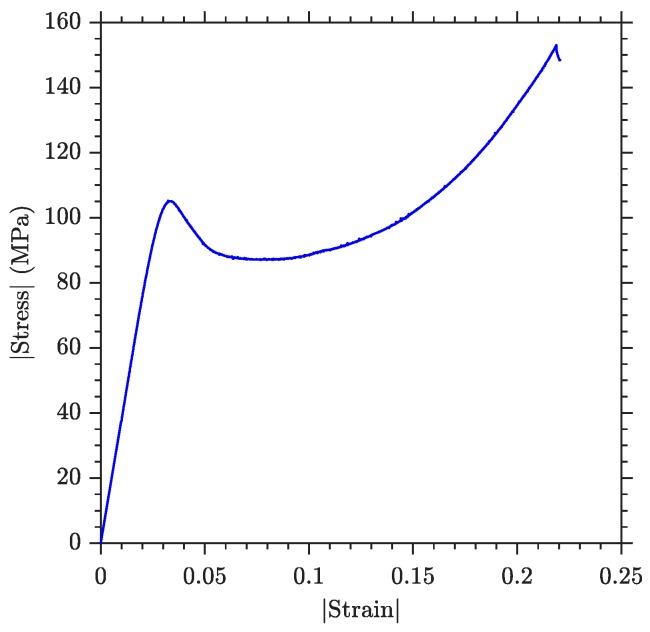
Compressive stress-strain behaviour of the material measured by cylinder compressive testing of blank specimen.

While the neat resin under tensile loading has a fragile linear behaviour, a standard uniaxial compression testing is here performed in order to determine the particular response of the blank epoxy under compression. A first stage of linear response can be observed (Figure 8), followed by a highly non-linear behaviour associated to plasticity. During the linear stage a compressive elastic modulus of 3.66 ± 0.08 GPa is observed, which agrees with the value obtained in the blank specimen under three-point bending (Table 4). The end of the linear elastic response is produced at a stress level of 105.90 ± 1.61 MPa and the compressive strength of the material at 146.45 ± 7.18 MPa.

During three-point bending testing the hypothesis of tensile promoted failure is ensured due to the fact that the maximum compressive stresses in the flexural specimens are 119.45 ± 6.18 MPa, a value that is lower than the compressive strength observed in the results of uniaxial testing. This maximum value of the compressive stress coincides with the onset of plastic deformation (Figure 8), indicating that the compressed region in flexural specimens is bearing force while plastic deformation occurs. This could be a source of the non-linearity that appears in the neat specimen at high levels of strain (Figure 6).

### 3.3. Numerical Results

**Figure 9:**
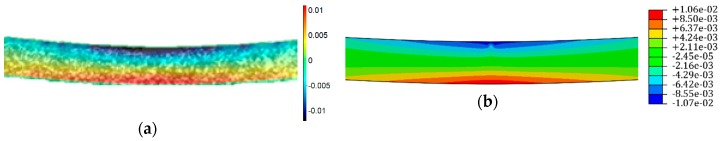
Comparison of strain contour maps obtained: (**a**) experimentally through DIC and (**b**) numerically by FEM.

Two numerical approaches were defined in previous chapter. Firstly, the linear elastic simulation, based on FEM, allows us to corroborate the adequacy of the analytical methodology based on the homogenised section technique. Figure 9 illustrates that there is good agreement between the experimental data and the numerical model, considering the deviation of the NF and the different tensile and compressive elastic moduli. This evidence confirms the necessity of considering the elastic mechanical behavior of the material, in order to reach accurate results when flexural loading is present. The choice of a linear elastic simulation to reproduce this loading scenario produces acceptable results, since the epoxy resin exhibits a general fragile behavior under bending, and therefore, the material works within the linear elastic region.

**Figure 10:**
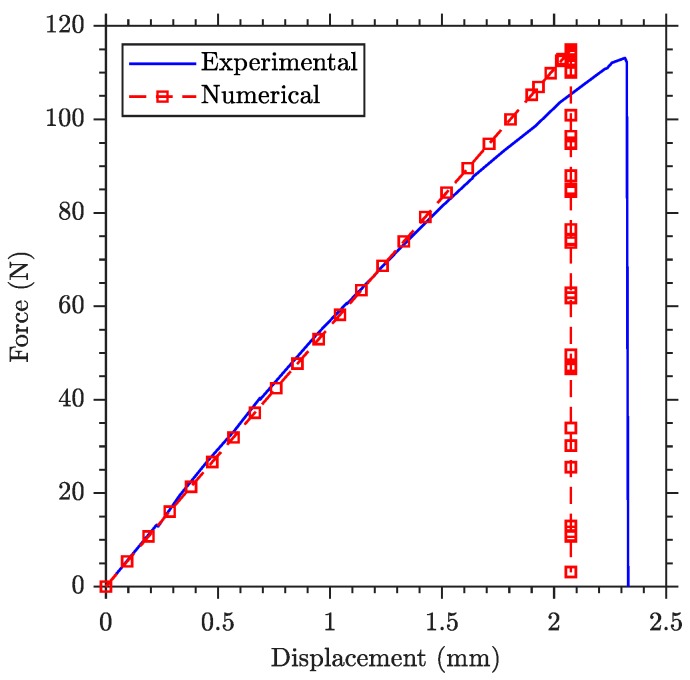
Comparison of applied force versus maximum deflection plot from eXtended finite element method (XFEM) simulations and three-point bending test of blank specimen.

Secondly, simulations using XFEM reproduce how the crack starts and propagates step-by-step, and they allow to extract information from each one of these iterations. The force-displacement results of this simulation are compared with the experimental data in Figure 10. First, good agreeement in rupture force is observed, with a small difference explained by dimensional deviation of manufactured specimen with regard to nominal values. Secondly, the XFEM simulation, based on a linear elastic material model, is not able to capture the non-linear behaviour due to polymer yielding, leading to a difference on maximum displacement reached at failure force. Additionally, the fracture energy applied to the simulation captures the brittle failure measured experimentally, obtaining a sudden force drop during crack propagation.

**Figure 11:**
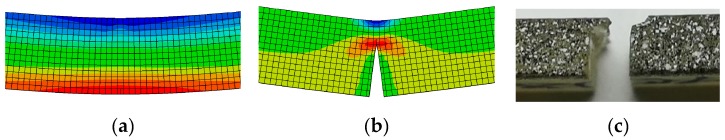
Normal strain contour plot from XFEM simulations corresponding to (**a**) crack initiation; and (**b**) end of propagation; and (**c**) experimental fracture.

The simulation using XFEM enable us to understand the experimentally observed crack. The application of a failure model, based on traction-separation, seems adequate because the crack propagates numerically starting at the maximum tensile stress point (Figure 11a). Then it spreads fragilely vertically until the cross-section is considerably reduced, thereby producing an area where tensile stresses at the edge of the crack cannot continue propagating the crack transversely (Figure 11b). This is due to the presence of compressive stresses near the singularity caused in the vicinity of the area where the load is applied. At this point, the simulation reaches a situation of difficult convergence. If the experimentally obtained crack is observed, the crack propagation reaches a scenario by which the end of the fracture is caused by abrupt instability. Finally, this leads the specimen to pivot around the load pin, producing a step-shape failure close to the upper face (Figure 11c).

### 3.4. Microscopic Examination and Fracture Surface Analysis

**Figure 12:**
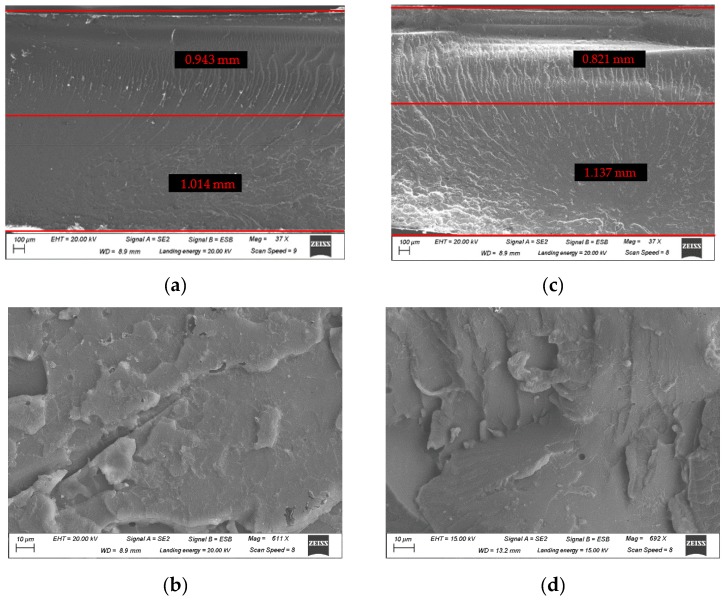
Scanning electron microscopy (SEM) fractographies: (**a**) and (**b**) neat resin; (**c**) and (**d**) 0.3 wt % GO reinforced epoxy.

Microscopic observation by means of Scanning Electron Microscopy (SEM), using Zeiss GeminiSEM 500, is performed to analyze the fracture surface. For ease of understanding, the orientation of the specimens in the fractographies ensures the notation is followed, i.e., the tensile loaded region is at the bottom of the picture. Figure 12a,c shows the three point-bending tested specimens made of blank epoxy, and the 0.3% GO reinforced material under low magnification, respectively. They exhibit a glassy and smooth fracture surface, typical of brittle thermoset polymers [48,49]. In both pictures at least three well-differentiated zones can be defined: starting from the bottom, the crack propagates easily through the tensile loaded region as characteristic of brittle crack initiation. In the mid region, feather markings develop while the fracture growths, with a higher crack forking related to the fast fracture energy liberation. Finally, we can observe again the step-shape fracture at the upper part of the specimen, related to the difficulty of crack propagation in the vicinity of the loading pin, where the maximum compressive stress arises.

An important distinction should be made between the neat and reinforced epoxy, as the second one shows a higher density of feather-markings. The high roughness of the GO reinforced specimen, i.e., the presence of deep line markings, demonstrates less brittle behavior, which makes us think of a higher fracture toughness due to the reinforcement. Although, specific fracture toughness testing should be performed to reach a firm conclusion, this idea agrees with most previous studies of graphene-derivative reinforced polymers [19,23,24,28].

In Figure 12a,c the presence of a zone, in which the river lines are scattered and curved, reveals a transition between the tensile and compressive loaded regions. The stress and strain distributions schemed in Figure 4 clarifies the idea of a transition between regions, a less defined failure mode is find close to the NF due to the lower values of the tensile and compressive stress and strain states. The dimensions of both regions can be estimated by considering the NF in the intermediate position of the transition region, which can be related to the strain measurements performed by means of the DIC system. The thicknesses ratios obtained for the fractographies (with the dimensions of *t* and *c* included in the pictures) are 1.08 and 1.41, while the measurements obtained via application of DIC during three-point bending testing are 1.09, and 1.38, for the neat resin and 0.3% GO reinforced epoxy, respectively. Moreover, Figure 12c illustrates the increase of the tensile loaded thickness *t* due to the 0.3 wt % GO reinforcement, that according to Equation (1) implies the increment of the compressive elastic modulus. This affirmation coincides with the rise shown in Figure 6, by which the slope of the stress-strain curved augments with the percentage of reinforcement.

Higher magnification SEM micrographies are shown in Figure 12b,d in order to analyze the dispersion and microstructural changes due to reinforcement. At this scale both specimens show little difference. The lack of particle aggreagates in the reinforced material is noticeable, demonstrating that there is good integration and dispersion of the reinforcement. We note the importance of the dispersion over the mechanical properties, and that the manufacturing method applied in this work avoids the use of solvents or energetic mixing methods, which leads to a easily scalable solution.

## 4. Conclusions

This work describes the mechanical behaviour of a reinforced epoxy resin with low GO content. Regarding the production of the reinforced material, a uniform dispersion of the nano-filler is obtained without requiring the use of solvents or sonication. This is explained by the affinity toward epoxy resin of the different functional groups in the GO and the preferential interfacial interaction between both components, spawned by the previous processing (dispersion, centrifugation, and lyophilisation) of GO.

The three-point bending tests results were analysed, together with the DIC and the analytical models, which describe the influence on the flexural response of the different elastic behaviours under tension and compression. This innovative procedure allows to appreciate clear non-linear tendencies in the tensile and compressive properties when increasing the percentage of reinforcement. The strength and stiffness are significantly modified when GO is applied as reinforcement. While, the tensile behaviour worsens very slightly, the compressive properties greatly improved, reaching up to approximately 39% increase of the compressive elastic modulus for the 0.3 wt% GO, compared with the neat epoxy.

Furthermore, changes in mechanical properties were related to the displacement of the NF, which results in a growth of the region submitted to tensile stresses when the reinforcement ratio is increased. Developing this idea, an analytical relationship, between the position of the NF and the percentage of reinforcement, has been described. Based on this mathematical model, further research into property optimization could be performed in future studies.

The compressive testing campaign validates the elastic properties obtained by flexural testing, also describing a possible source of the non-linearity perceived just before the final failure in the specimens tested under bending. This non-linearity is due to the yielding behaviour of the resin under compressive loads.

In relation to the numerical analyses, static linear elastic simulations, using FEM validates the experimental results, depicting the non-symmetric stress/strain distribution, in agreement with the homogenised section technique applied to the analysis of bending testing data. Additionally, crack propagation simulations, using XFEM, offer a good basis of the brittle failure produced in the flexural tests and, most importantly, ensure the breaking force is estimated accuratley. The addition of plasticity material models could help improve the numerical results in future works.

## Figures and Tables

**Table 1:** Composition of studied materials.

Material Denomination	Composition	
Blank	100.0 wt % epoxy	-
0.1 wt % GO	99.9 wt % epoxy	0.1 wt % GO
0.2 wt % GO	99.8 wt % epoxy	0.2 wt % GO
0.3 wt % GO	99.7 wt % epoxy	0.3 wt % GO
0.5 wt % GO	99.5 wt % epoxy	0.5 wt % GO
0.3 wt % Graphite	99.7 wt % epoxy	0.3 wt % Graphite

**Table 2:** Material properties for the blank epoxy used in the simulation.

*E*_t_ (GPa)	*E*_c_ (GPa)	*ν*	$\sigma_{f}(\mathrm{MPa})$	$\varepsilon_{f}$	$G_{IC}$ (kN/m)
3.10	3.63	0.43	110.43	0.05	27.6

**Table 3:** Tensile strength and maximum compressive stress measured via bending testing.

Material	*σ*_t_ (MPa)	*σ*_c_ (MPa) ^1^
Blank	110.43 ± 5.68	119.45 ± 6.18
0.1 wt % GO	100.52 ± 3.30	121.67 ± 4.37
0.2 wt % GO	97.41 ± 3.66	122.78 ± 4.45
0.3 wt % GO	94.50 ± 2.93	123.15 ± 4.28
0.5 wt % GO	75.90 ± 5.44	103.70 ± 7.22
0.3% Graphite	88.23 ± 4.92	103.83 ± 5.80

^1^ Note that these values are the maximum compressive stress measured, not the compressive strength.

**Table 4:** Tensile, compressive and flexural elastic moduli measured via bending testing.

Material	*E*_t_ (GPa)	*E*_c_ (GPa)	*E*_f_ (GPa)
Blank	3.10 ± 0.04	3.63 ± 0.05	3.35 ± 0.04
0.1 wt % GO	3.06 ± 0.02	4.46 ± 0.04	3.66 ± 0.03
0.2 wt % GO	3.00 ± 0.04	4.78 ± 0.07	3.74 ± 0.05
0.3 wt % GO	2.96 ± 0.04	5.04 ± 0.06	3.79 ± 0.04
0.5 wt % GO	2.81 ± 0.04	5.20 ± 0.10	3.72 ± 0.08
0.3% Graphite	3.01 ± 0.03	4.17 ± 0.04	3.52 ± 0.03
